# Collaboration of Japanese Kampo Medicine and Modern Biomedicine

**DOI:** 10.1155/2014/646947

**Published:** 2014-06-11

**Authors:** Kenji Watanabe, Gregory A. Plotnikoff, Takeshi Sakiyama, Heidrun Reissenweber-Hewel

**Affiliations:** ^1^Faculty of Environment and Information Studies, Keio University, Fujisawa, Kanagawa 252-0882, Japan; ^2^Penny George Institute for Health and Healing, Allina Health, Minneapolis, MN 55407, USA; ^3^Ishikawa Clinic, Hacchobori, Chuo, Tokyo 104-0032, Japan; ^4^Technical University of Munich and Private Clinic for Japanese Medicine, Gräefelfing, 82166 Munich, Germany

Worldwide, health care systems are increasingly highlighting the importance of traditional medicines. The World Health Organization (WHO) plans to introduce traditional medicine into the international classification of diseases (ICD) for the first time since it started in 1900. Kampo medicine is a traditional Asian medical system that is unique in many ways. Kampo was transferred from the ancient Han Chinese dynasty and uniquely developed in Japan, especially during the Edo period (1603–1867). The theoretical understanding and the use of the abdominal examination “Fukushin” to assess the patient's constitutional state are particular to Kampo.

After the Meiji restoration in 1867, Japan's new government accepted only Western medicine from Europe and founded one medical licensure system. The result was suppression of acquired wisdom and insights with marginalization of practitioners until 1976. At that time, the Japanese Medical Association promoted its coverage by Japan's National Health Insurance program by physicians who were trained in Western medicine. And now, all of Japan's 80 medical schools teach Kampo medicine. As a result, roughly 90% of physicians use Kampo medicine in their daily practice. This is a very unique model of integration of traditional medicine and modern biomedicine. To better understand the promise of this integration, this special issue features Kampo medicine in the context of modern biomedicine.

Many provocative articles are included in this special issue. To begin, K. Katayama et al. address the current situation of Kampo use in the National Health Care program. The authors analyzed 67,113,579 health care claim records and found that only 1.34% represented Kampo prescriptions. This suggests that a very small portion of conventional practice includes Kampo treatment even though many physicians use Kampo. H. Okamoto et al. present us with a case series of patients with refractory glossodynia. Among 39 patients, 69.2% of patients reported a beneficial effect. This is one of the examples in which Kampo treatment is effective even for the difficult cases in the Western biomedicine. K. Ogawa et al. show the usefulness of daiobotanpito for the treatment of acute diverticulitis. Y. Tanaka and T. Sakiyama report the case series of the usefulness of the Kampo treatment for pediatric emotional and behavioral disorders which were also refractory to the modern biomedicine. M. A. Bahar et al. reported that goshajinkigan prevents paclitaxel induced peripheral neuropathy without interfering with the anticancer action of paclitaxel in the basic research. Kampo medicines are often used for the purpose of the reduction of the side effects of chemotherapy for malignancies. K. Watanabe et al. report the potentially preventive effect of diabetic complications. Goshajinkigan is often used for the neuropathy from diabetes mellitus. Additionally, this Kampo medicine may be beneficial for the blood glucose control. K. Katayama et al. report a computer-based diagnostic way of Kampo patient patterns termed “Sho.” This represents a promising blend of modern technology for a new world of traditional medicine in the future. Y.-C. P. Arai et al. reported about Fukushin, Kampo's unique diagnostic procedure. Certain Fukushin findings are related to the anxiety-depression levels. T. Namiki et al. report that cytosine-adenine (CA) repeat polymorphism of the estrogen receptor *β* gene can be the predictive biomarker of the effectiveness of Kampo medicine for the treatment of the climacteric syndrome. T. Yoshino et al. and H. Tokunaga et al. describe* hie *(cold sensation) and* hiesho *(cold disorder). Hie and hiesho are very important concepts in Kampo treatment. T. Yoshino et al. characterized hie and hiesho. H. Tokunaga et al. characterized the differences of male hie, female hie with menstruation, and female hie after menopause by data mining method. To conclude, S. Yakubo et al. summarize the history and pattern diagnosis of Kampo medicine.

Together, these articles represent the promise and challenges present in the scientific understanding of Japan's herbal medicine tradition. We hope these articles help the readers to understand and appreciate the potential power of Kampo medicine outside of Japan. We invite you to explore Kampo.

*Kenji Watanabe* *Kenji Watanabe* *Gregory A. Plotnikoff* *Gregory A. Plotnikoff* *Takeshi Sakiyama* *Takeshi Sakiyama* *Heidrun Reissenweber-Hewel* *Heidrun Reissenweber-Hewel*

